# Discovery of novel cholesteryl ester transfer protein (CETP) inhibitors by a multi-stage virtual screening

**DOI:** 10.1186/s13065-024-01192-5

**Published:** 2024-05-03

**Authors:** Yanfeng Liu, Liangying Deng, Feng Ding, Qiang Wang, Shuran Zhang, Nana Mi, Wenhui Zhang, Bailin Zeng, Huangjin Tong, Lixing Wu

**Affiliations:** 1 Nanjing Lishui District Hospital of Traditional Chinese Medicine, Nanjing, China; 2 Affiliated Hospital of Integrated Traditional Chinese and Western Medicine, Nanjing University of Chinese Medicine, Nanjing, China; 3 School of Basic Medicine and Clinical Pharmacy, China Pharmaceutical University, Nanjing, China

**Keywords:** Cholesteryl ester transfer protein, 3D-QSAR pharmacophore modeling, Molecular docking, Molecular dynamics simulation, Virtual screening

## Abstract

Cholesteryl ester transfer protein (CETP) is a promising therapeutic target for cardiovascular diseases. It effectively lowers the low-density lipoprotein cholesterol levels and increases the high-density lipoprotein cholesterol levels in the human plasma. This study identified novel and highly potent CETP inhibitors using virtual screening techniques. Molecular docking and molecular dynamics (MD) simulations revealed the binding patterns of these inhibitors, with the top 50 compounds selected according to their predicted binding affinity. Protein–ligand interaction analyses were performed, leading to the selection of 26 compounds for further evaluation. A CETP inhibition assay confirmed the inhibitory activities of the selected compounds. The results of the MD simulations revealed the structural stability of the protein–ligand complexes, with the binding site remaining significantly unchanged, indicating that the five compounds (AK-968/40709303, AG-690/11820117, AO-081/41378586, AK-968/12713193, and AN-465/14952302) identified have the potential as active CETP inhibitors and are promising leads for drug development.

**Graphical Abstract:**

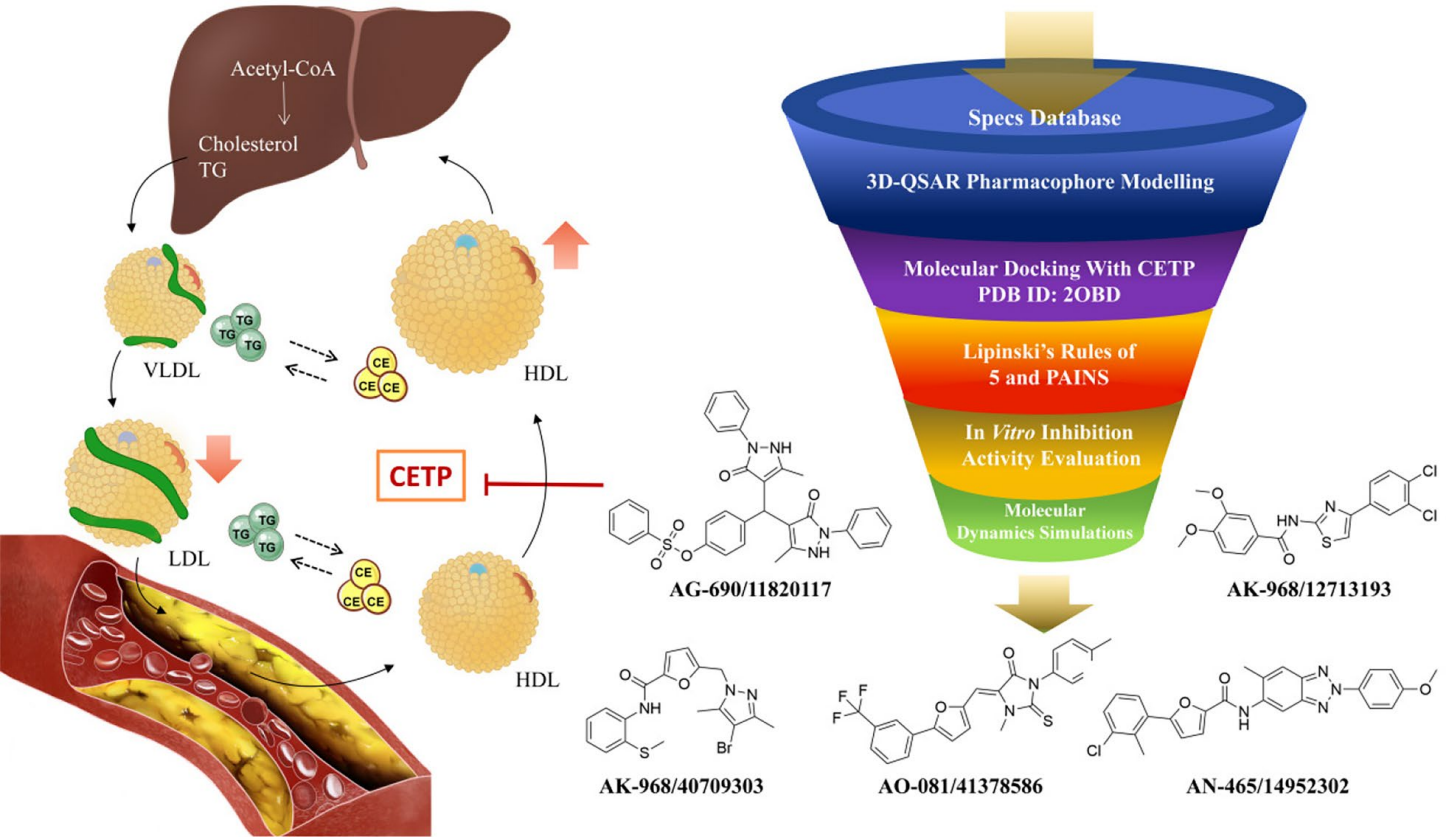

**Supplementary Information:**

The online version contains supplementary material available at 10.1186/s13065-024-01192-5.

## Introduction

Cholesteryl ester transfer protein (CETP) is a single-chain protein comprising 476 amino acid residues [1]. It is predominantly found in the plasma and interacts with lipoproteins, including high-density lipoproteins (HDLs) and low-density lipoproteins (LDLs) [2]. CETP comprises four domains: N-terminal signal peptide, N-terminal β-helix, C-terminal β-lobe, and C-terminal tail, which form a three-dimensional structure [3, 4]. In addition, it has a flexible conformation and can form a continuous tunnel through its long axis, enabling the directional transfer of cholesteryl ester and triglycerides. The n-terminal β-barrel structure penetrates the HDL surface to promote cholesterol ester uptake [3, 5]. A previous structural analysis of the CETP [6] proposed three models: shuttle, tunnel, and dimer tunnel. According to the shuttle model, CETP binds more strongly to HDL than LDL. Due to the higher pressure on the HDL end, the cholesteryl esters move toward the LDL end and bind to LDL, facilitating their transportation into the bloodstream and accelerating atherosclerosis. However, when CETP inhibitors enter the CETP channel, they increase the rigidity of CETP binding to HDL, increasing the number of free CETP molecules that bind exclusively to HDL. Consequently, the transfer of cholesterol esters from HDL to LDL is reduced, thereby decreasing the amount of cholesterol esters transferred into the bloodstream, ultimately slowing atherosclerosis. Thus, CETP inhibition lowers the LDL-C levels, increases the HDL-C levels, and maintains cholesterol homeostasis. Among the currently developed CETP inhibitors, dalcetrapib, anacetrapib, and their binding modes are worth investigating. They bind to different sites on CETP and induce conformational changes in the molecule associated with reduced residual CETP activity [7, 8]. However, in clinical trials, dalcetrapib did not increase the HDL-C levels. Although anacetrapib cannot be used in practical applications because of its lipid solubility, a previous study showed that it can simultaneously elevate the HDL-C and LDL-C levels [9]. Therefore, based on the available findings, we believe that the binding mode of anacetrapib to CETP holds greater potential than that of dalcetrapib for developing new CETP inhibitors. Several studies have utilized molecular docking techniques to investigate the binding patterns and affinity of CETP with potential inhibitors [10–12]. These studies use molecular docking to provide insights into their binding sites and modes. However, in this study, we combined molecular docking and other computational methods to screening the poteintial compounds.

Therefore, this study aimed to screen the Specs databases to identify potential compounds that target CETP. We utilized pharmacophore modeling and molecular docking techniques to search for CETP inhibitors, while implementing Lipinski's Rule of Five and pan-assay interference compound (PAINS) filters. The developed models successfully identified highly potent lead compounds that were experimentally validated for activity and efficacy. Finally, molecular dynamics (MD) simulations were performed to investigate the interplay between the screened compounds and their targets, elucidate the intricate details of their interactions, and provide insights into their binding mechanisms. These findings suggest effective strategies for developing lead-based CETP inhibitors.

## Materials and methods

### 3D-quantitative structure–activity relationship (QSAR) pharmacophore modeling

#### Compound preparation

A 3D-quantitative structure–activity relationship (QSAR) pharmacophore model was developed to study the relationship between chemical substances and their biological activities [13]. The active compound selection was crucial in this study. Previous studies identified 40 compounds with similar biological activities [2, 14, 15]. ChemDraw was used to map the structures of the selected CETP inhibitors, which were saved as.sdf files. The 2D structures of these compounds are presented in Table 1 and 2. Discovery Studio (DS) was used to convert the selected compounds into a 3D conformation with energy minimization and optimization using the Merk molecular force field (MMFF). These conformations were used to construct pharmacophores and predict the activities of the compounds in the database.

**Table 1:**
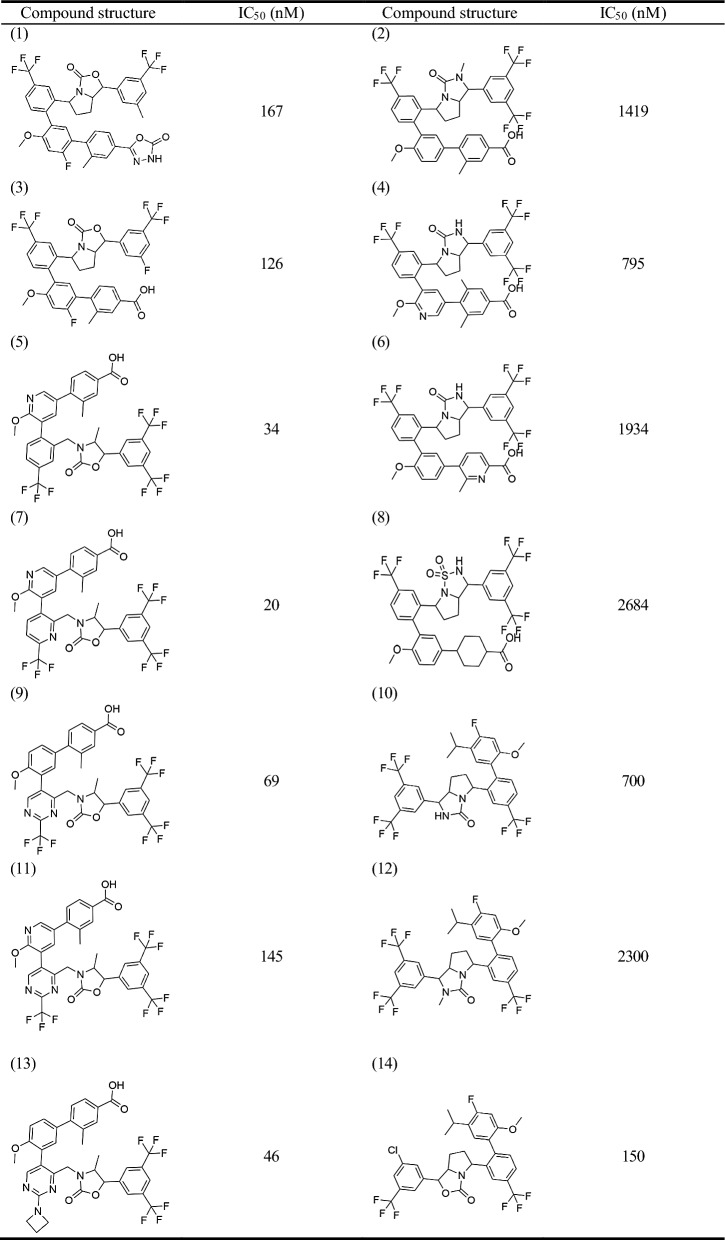

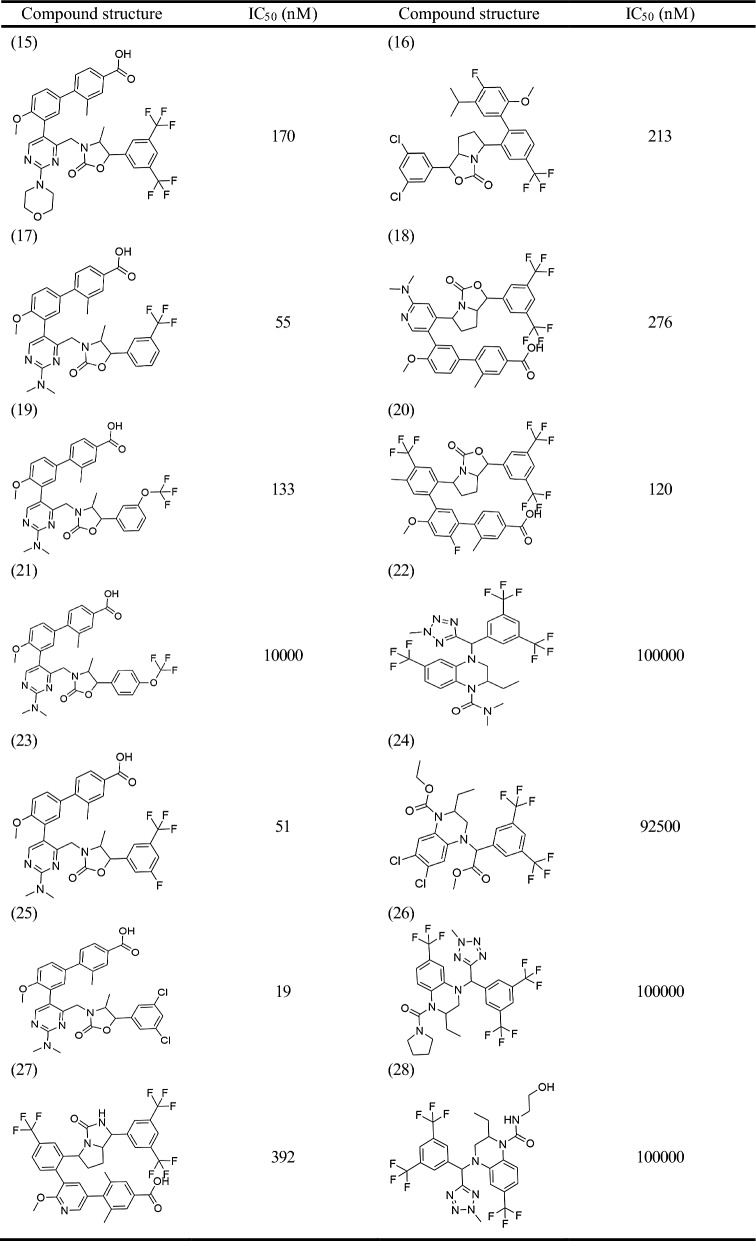
2D structure of the training set

**Table 2:**
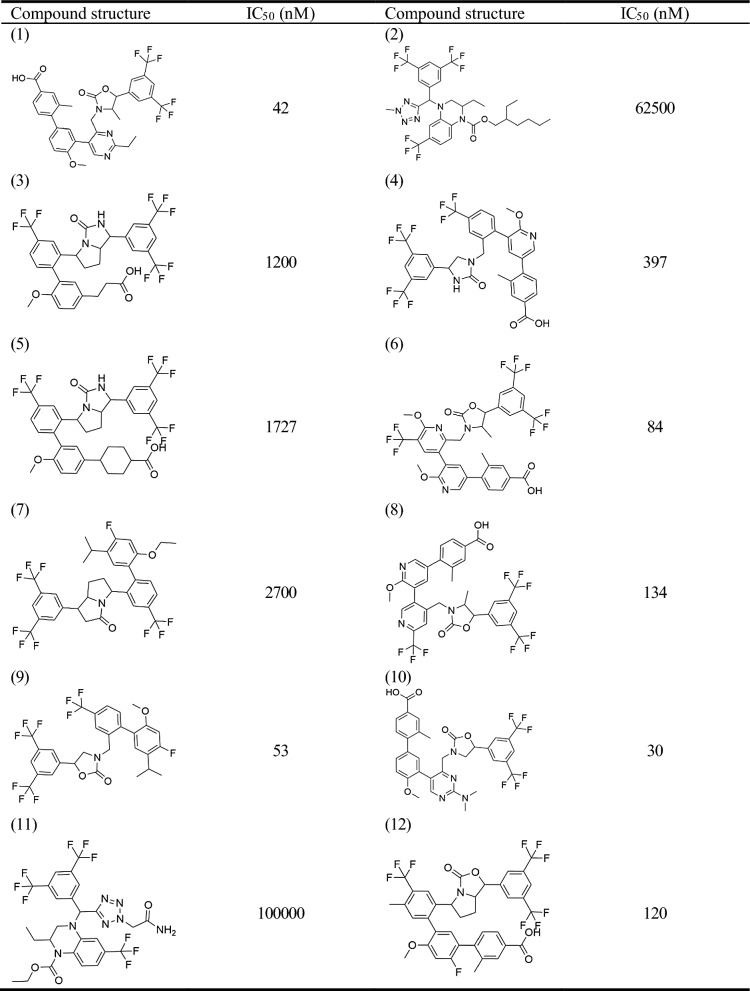
2D structure of the test set

QSAR aims to quantitatively capture the structure–activity relationships between small molecule structures and their activities. For model construction, we sourced compounds targeting the same biological target from Binding Database (BindingDB) and other publications, with each compound having an activity concentration range of over four orders of magnitude. Prior to modeling, we performed molecular alignment of the shared pharmacophore groups. The compounds were divided into independent training and test sets. The training dataset was used to build the pharmacophore hypothesis, whereas the test set was used to cross-validate the resultant QSAR model, adhering to the standards for rigorous QSAR model development [16]. Based on the half-maximal inhibitory concentration (IC_50_) values, the training and test set compounds were classified into four classes: most active (pIC_50_ ≤ 2), active (2 < pIC_50_ ≤ 3), moderately active (3 < pIC_50_ ≤ 4), and inactive (pIC_50_ > 4) [16]. By accounting for activity variability, conformational alignment, and independent evaluation, our approach aims to derive statistically robust and predictive QSAR relationships for the target of interest.

#### Constructing the 3D-QSAR pharmacophore modeling

The feature-mapping module in DS was used to identify the pharmacophore features of the two most active compounds. The identified features included hydrogen bond acceptors, hydrophobicity, negative ions, and ring aromaticity. A 3D-QSAR pharmacophore was constructed in the DS using 28 training set compounds, generating multiple conformations in the best mode. Ten pharmacophore models were developed based on statistical parameters, such as total cost, cost difference, maximum fit, features, root mean-square deviation (RMSD), and correlation. Each compound generated 255 conformations with an energy constraint of 10 kcal/mol, a minimum interference distance of 1.5, and an IC_50_ for selecting the activity data.

#### Verifying the pharmacophore and database screening

The top-ranked model was selected and tested using two methods: test set validation to evaluate predictive power and Fischer's randomization test to assess reliability [16]. In Fischer randomization validation, compound activities are randomly permuted to dissociate structures from their original measured activities, thereby disrupting the inherent structure–activity relationships. Multiple pharmacophore models were constructed from these randomized datasets, and their fitting performances were compared with those of the original model. The present study used the Specs molecular database (https://www.specs.net/) for screening because it is a leading source for molecular information offering a diverse range of molecules, abundant data, and various scaffold types. It provides extensive data on molecular structures and chemical properties, enabling in-depth research and analysis. Compared to other databases, Specs database offers broader data coverage and higher data quality assurance. In addition, they are easily accessible, making them convenient for researchers in molecular biology and chemistry. The ligand-based pharmacophore mapping feature of DS was then used as a 3D query to screen the Specs database using the well-validated 3D-QSAR model Hypo1. Compounds that matched the mapped pharmacophores and the molecules with predicted activity levels below 1 μM were retained during the screening process.

### Molecular docking and hit selection

The binding site of the CETP structure (PDB ID:2OBD) was docked with the compounds identified using a pharmacophore-based screening procedure. The binding-site prediction was performed using ProteinsPlus (https://proteins.plus/pages/about) [17]. The structure-based virtual screening of the database against CETP was performed using AutoDock Vina (Scripps Research Institute, California) [18]. Molecular docking was performed using the Lamarckian algorithm. All water molecules from the CETP structures were removed before performing molecular docking calculations. Subsequently, hydrogen atoms were added, and the Gasteiger partial charges were assigned. Before the docking of the drug-like molecules, the reliability of the AutoDock Vina docking software was evaluated. The screening power was similar to the scoring power; however, the ability of the programs to identify known binders seeded in large databases of non-binders or decoys was measured. The assessment of virtual screening outcomes involved estimating their enrichment levels and analyzing the receiver operating characteristic (ROC) curve [19, 20]. The concentrations of the active compounds in the virtual screening results were used as benchmarks to determine the enrichment factor. ROC curve analysis is effective in evaluating the precision of virtual screening outcomes. In addition, they can distinguish between active and inactive molecules and establish a definitive boundary between the two groups [21].

Ten docking poses were created for each compound, and the pose with the lowest binding affinity was chosen as the best-hit compound. The receptor–ligand complexes with the lowest binding affinities were further analyzed. Subsequently, to eliminate potentially problematic compounds, Lipinski's Rule of Five and PAINS screening were conducted using ADMETlab 2.0 [22] and SwissADME [23]. The physicochemical properties of the 26 compounds, calculated using SwissADME [23] and ADMETlab 2.0 [22] are shown in Additional file 1: Table S1.

### Biological evaluation

The anti-CETP activity of the molecules was measured using a standard fluorescent CE-transfer assay (CETP Inhibitor screening kit [ab283403]; Abcam, Cambridge, UK). Briefly, the compounds were purchased from Bidepharm (Shanghai, China), fully dissolved in dimethyl sulfoxide (Sigma–Aldrich, St. Louis, MO, USA), and stored in a nitrogen-filled cabinet. A solution without recombinant CETP (rCETP) served as the background. The positive controls contained rCETP but no test compounds. In the assay buffer, the donor (4 mL), acceptor (4 mL), and test drugs (1 mL) were mixed with 30 ng rCETP (200 mL). The fluorescence intensity was measured in kinetic mode using a fluorimeter (Agilent BioTek Synergy H1 Multimode Reader) at Ex/Em = 480/511 nm. The inhibition ratio was computed after a 30-min incubation at 37 °C. The results of the CETP inhibition assay are presented in Additional file 1: Table S2. Furthermore, graphical representations of the data were generated using GraphPad software.

### MD simulations

After conducting the CETP inhibition study, MD simulations were performed on the top five identified compounds. MD simulations via the GROningen MAchine for Chemical Simulations (GROMACS 2022.3) were used to evaluate the stability of the complex created between the target protein and the docked ligand in a dynamic environment [24–26]. A generation Amber force field (GAFF) [27, 28] was added to the small molecules using AmberTools22, whereas the protonation state of titratable residues was determined using the PDB2PQR server [29]. The simulation parameter files for CETP were generated using Amber99sb-ildn [30]. Next, the protein–ligand complex was hydrated using the TIP3P system throughout the simulation run, and counter ions were used to neutralize the simulation box. Energy minimization of the simulation system was achieved using the steepest descent method. Consequently, the canonical ensemble and constant temperature-constant pressure ensemble were used. Molecular simulations were conducted under periodic boundary conditions to reduce the edge effects. Finally, the system was subjected to a 100-ns production MD run with a timeframe of 2 fs. After completing the simulation, a built-in software tool was employed to assess the trajectory data. The tool calculates parameters like RMSD, root-mean-square fluctuation (RMSF), and protein rotation radius across each amino acid trajectory. The results were then combined with additional data, such as free energy (MMPBSA), for further analysis [31].

## Results and discussion

### Pharmacophore model generation and virtual screening

**Fig. 1:**
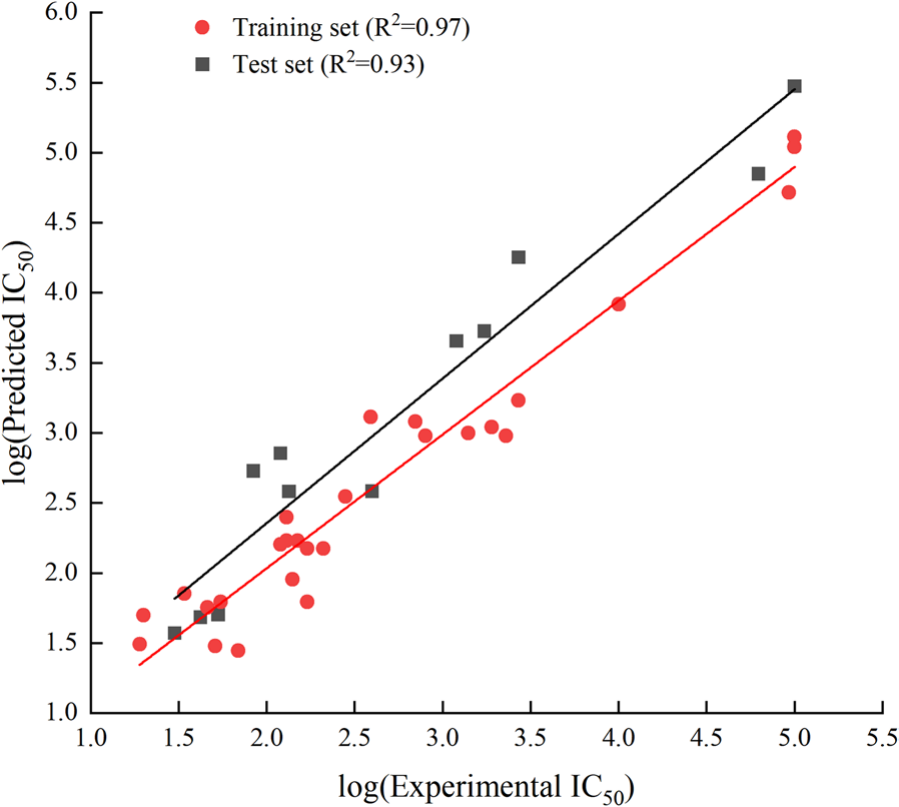
Graphical representation of the correlation between experimental and predicted activity values in logarithmic scale for training and test set compounds based on Hypo1

**Fig. 2:**
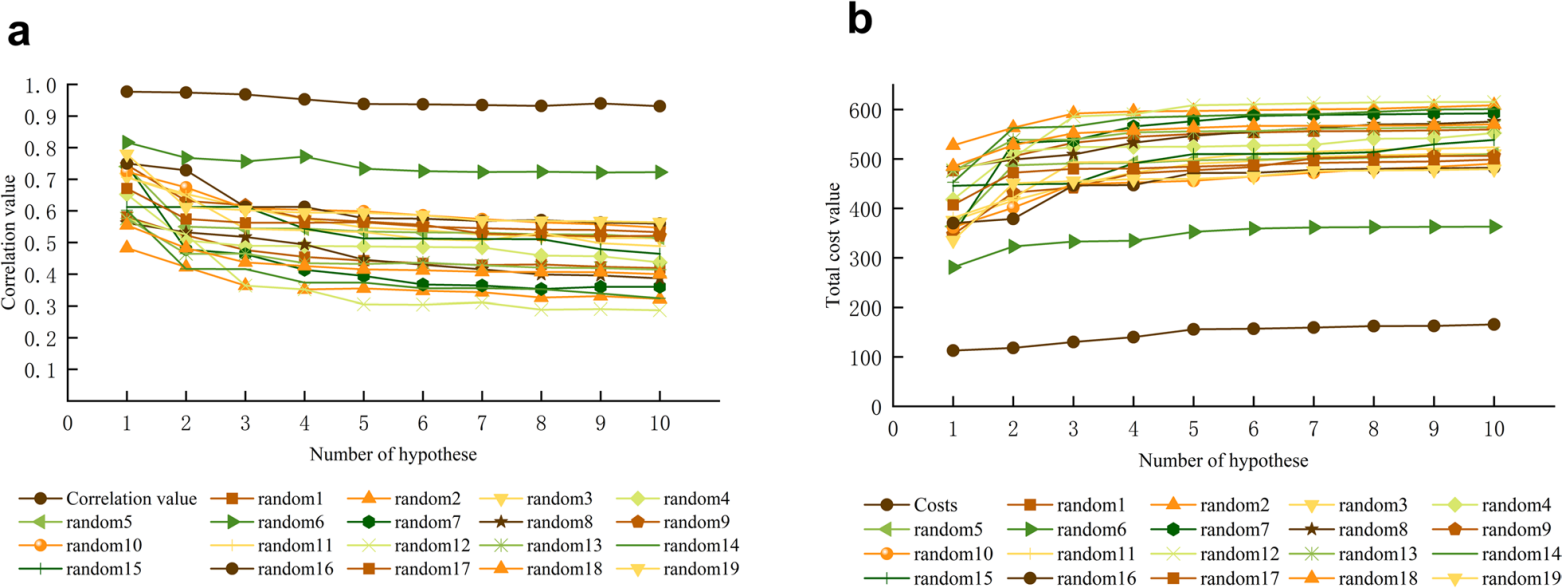
Graphical depiction of the total cost analysis and correlation of the initial spreadsheet and 19 random spreadsheets during Fischer’s randomization run. A confidence threshold of 95% was applied. **A** Correlation value and **B** total coat value

The training set of 28 compounds (Table 1) with diverse active values (most active (pIC_50_ ≤ 2), active (2 < pIC_50_ ≤ 3), moderately active (3 < pIC_50_ ≤ 4), and inactive (pIC_50_ > 4)) was used to construct the pharmacophore model. The top 10 developed pharmacophore hypotheses are summarized in Table 3, with the best model selected based on the total cost, cost difference, RMS, correlation, and maximum fit. Furthermore, all the generated hypotheses contained a hydrogen bond acceptor, indicating that it is essential for CETP inhibition. Hypothesis 1 was characterized by a maximum cost difference of 527.58, highest correlation value of 0.97723, lowest RMSD value of 1.35912, highest Q^2^ value of 0.945 and higer Q^2^_ext_ value of 0.8106 (Table 3). A lower total cost value indicates a higher level of matching and hence a more predictive pharmacophore model. A correlation coefficient is a statistical measure of the degree of association between two sets of quantitative variables. A higher correlation coefficient indicates the strongest correlation between variables. The RMSD reflects the atomic fluctuations of a system over a given period. A lower RMSD value indicates a higher stability of the protein–ligand complex during that period, indicating a more stable conformation. For an acceptable model, the value of Q^2^ and Q^2^_ext_ should be greater than 0.5. Hypothesis 1 also contained one hydrogen bond acceptor, three hydrophobic features, and one ring aromaticity feature, ranking it the best model. Assessing the pharmacophore model is critical for identifying reliable hit compounds for further applications. The test set (Table 2) was validated using 12 structurally different compounds. Hypothesis 1 indicated significant correlations between the predicted and actual biological activities of the training (R^2^ = 0.97) and test sets (R^2^ = 0.93) (Fig. 1). Fisher’s randomization test was used to evaluate the statistical significance of the HipHop model. A hypothesis was generated using 19 randomized spreadsheets with a confidence level of 95% (Fig. 2). The ligand-based pharmacophore mapping function of the DS resulted in mapping all aspects of the model to 484 molecules.

**Table 3:** Statistical details of 10 HypoGen algorithm-generated pharmacophore hypotheses

Hypo	Total cost	ΔCost^a^	RMSD^b^	Correlation	Max fit	Features^c^	Q^2^	Q^2^_ext_
1	113.056	527.58	1.35912	0.977230	11.0334	HBA\3HY\RA	0.945	0.8106
2	118.203	522.43	1.44329	0.974314	13.0794	HBA\3HY\RA	0.939	0.5686
3	130.353	510.28	1.60896	0.968104	12.5679	HBA\3HY	0.940	0.7777
4	140.092	500.54	1.94334	0.952864	8.91261	2HBA\3HY	0.886	0.5461
5	155.967	484.67	2.21801	0.938131	10.7763	HBA\3HY\RA	0.879	0.8127
6	157.167	483.47	2.23903	0.936911	10.3062	HBA\3HY\RA	0.867	0.8035
7	159.647	480.99	2.27600	0.934738	9.13603	2HBA\3HY	0.864	0.6912
8	162.636	478.00	2.32446	0.931826	10.3773	HBA\3HY\RA	0.843	0.8763
9	162.742	477.89	2.19205	0.93985	12.9834	HBA\2HY\RA	0.865	0.5297
10	165.845	474.79	2.33925	0.930965	10.5767	HBA\2HY\RA	0.765	0.2393

a:ΔCost is the difference between the null cost (640.633) and the total cost, b: RMSD is root mean square deviation. c HBA, hydrogen bond acceptor; HY, hydrophobic; RA, ring aromatic

### Molecular docking and drug-likeness analysis

**Fig. 3:**
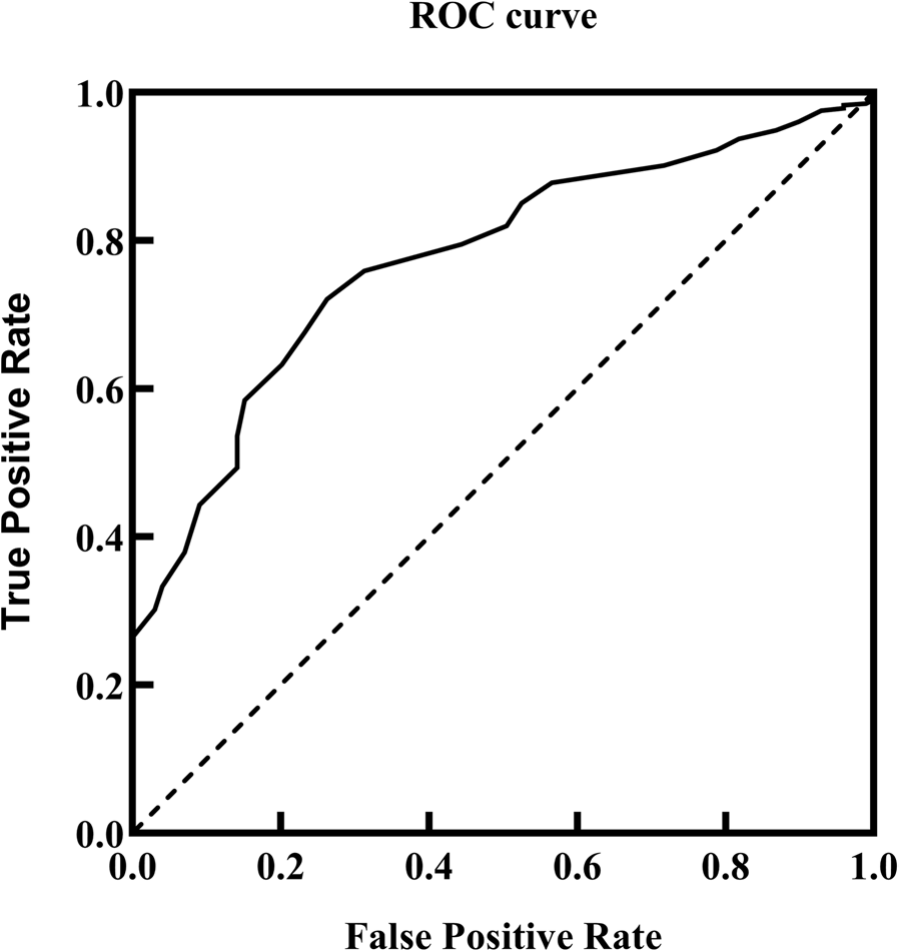
Receiver operating characteristic** (**ROC) curve for molecular docking efficiency validation

First, molecular docking analysis was conducted to investigate the binding modes of the screened virtual hits. The top-hit compounds identified by the pharmacophore-based virtual screening were docked to CETP protein (PDB ID:2OBD) [1]. The reliability of the AutoDock Vina docking software was evaluated before docking the selected molecules. In molecular screening, the decoy molecules are defined as compounds that bear physicochemical characteristics comparable to those of the active agents but fail to produce any activity with the target. Screening efficacy was assessed using two metrics: enrichment factor and ROC curves. The CETP inhibitors were collected from the ChEMBL database, and 1,548 decoy molecules were generated [32]. The center of the binding pocket was set at 12.461, 4.223, and 39.178, and its size was 33.75 Å × 40.5 Å × 42 Å. The area under the ROC curve (0.775) indicated the proficient performance of our molecular docking model in virtual screening. The enrichment factor for the first 2% was 2.02 (Fig. 3). After successful validation, the compounds selected from pharmacophore-based virtual screening were subjected to docking studies using CETP as a substrate. Subsequently, the compounds were evaluated based on their binding free energies and ranked in the order of importance. Finally, to identify the protein–ligand interactions, the PLIP algorithm, which employs a rule-based system of geometric constraints to match the interacting atoms, was used to measure the distances and angles among the atoms.

The drug-like properties essential for a compound to be considered a potential drug were evaluated. The top 50 compounds ranked by binding affinity were selected, and those that complied with Lipinski's Rule of Five [33] were retained for further investigation after the removal of PAINS (Additional file 1: Table S1) [34]. The analysis of the protein–ligand interactions was performed using the protein–ligand interaction profiler (PLIP) package [35]; the 3D presentations of the binding mode between CETP and the hits are shown in Additional file 1: Fig S1. Finally, 26 compounds were selected for subsequent biological evaluation. The binding affinities (kcal/mol) of the 26 compounds are listed in Additional file 1: Table S2.

### CETP inhibition study

**Fig. 4:**
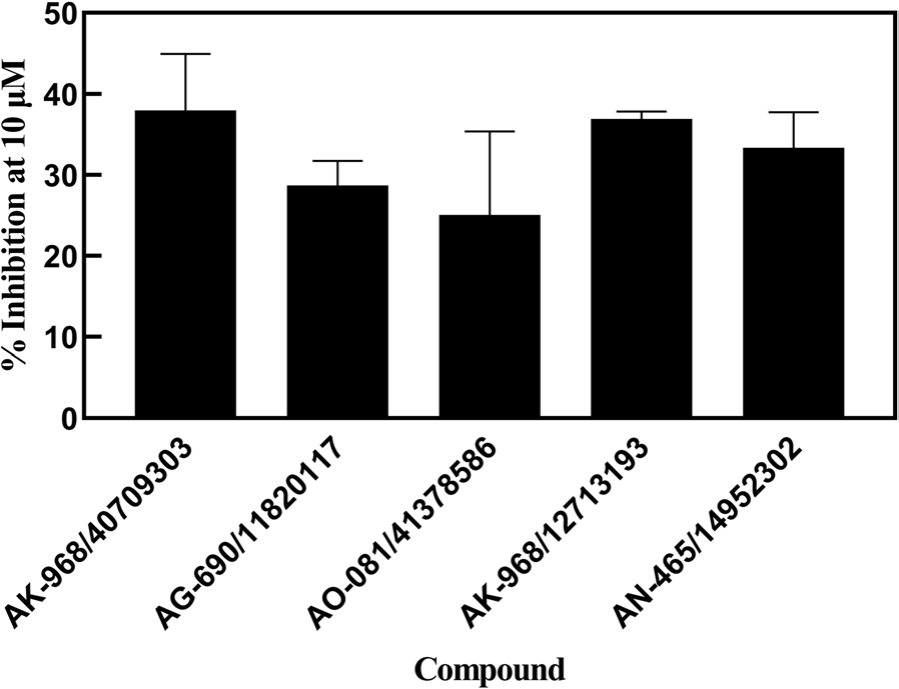
Screening assay of identified compounds as novel cholesteryl ester transfer protein (CETP) inhibitors in vitro. Inhibitory activity of the five potential inhibitors against CETP

All new molecules were purchased to evaluate their inhibitory effects against CETP using the CETP Inhibitor screening kit (fluorimetric). In vitro screening assay showed that five of the 26 compounds moderately inhibited human CETP activity (Fig. 4, Additional file 1: Fig S2 and Additional file 1: Table S3). These compounds, AK-968/40709303, AG-690/11820117, AO-081/41378586, AK-968/12713193, and AN-465/14952302, were considered potential lead compounds for the design of more effective CETP inhibitors.

### MD simulations

**Fig. 5:**
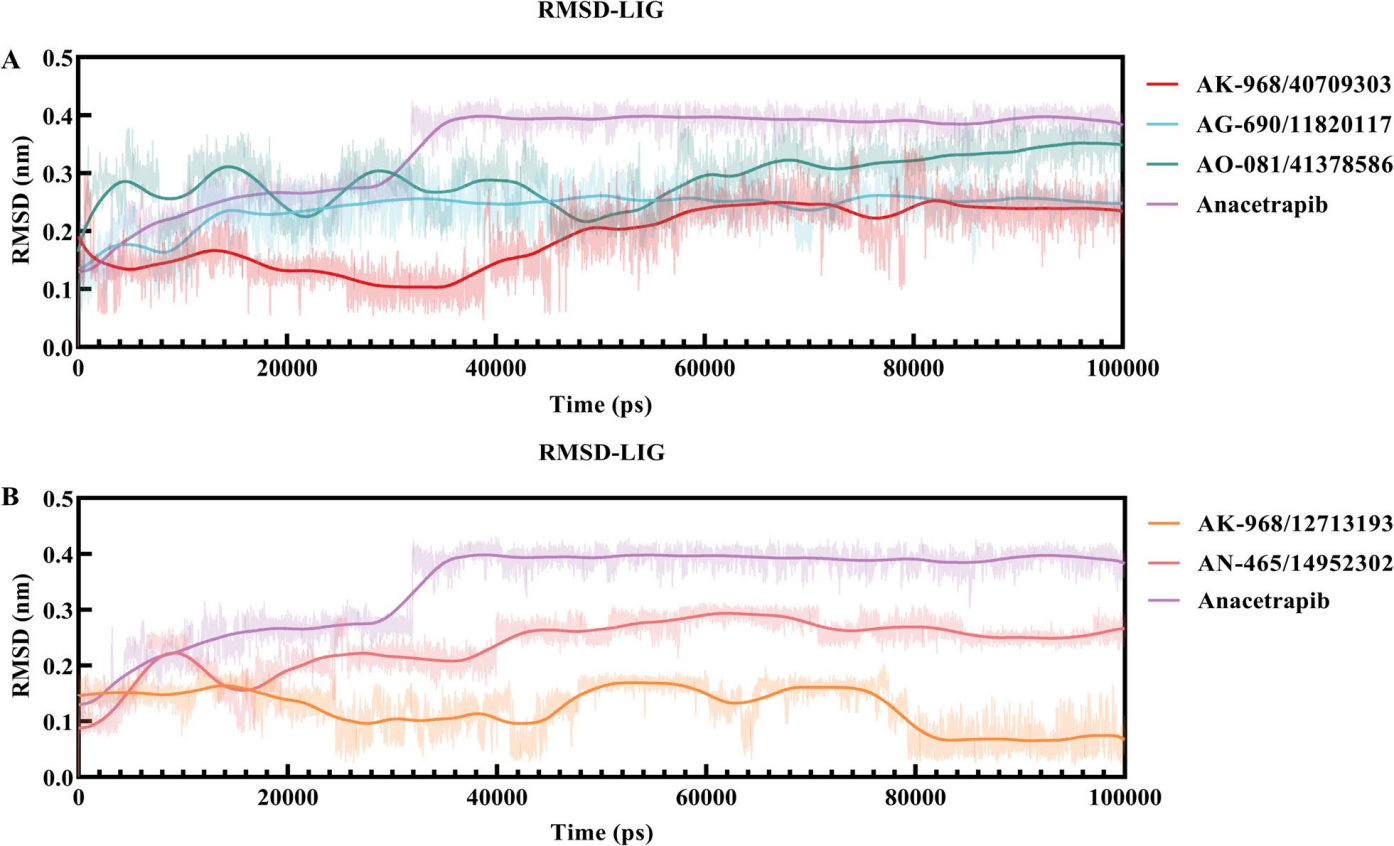

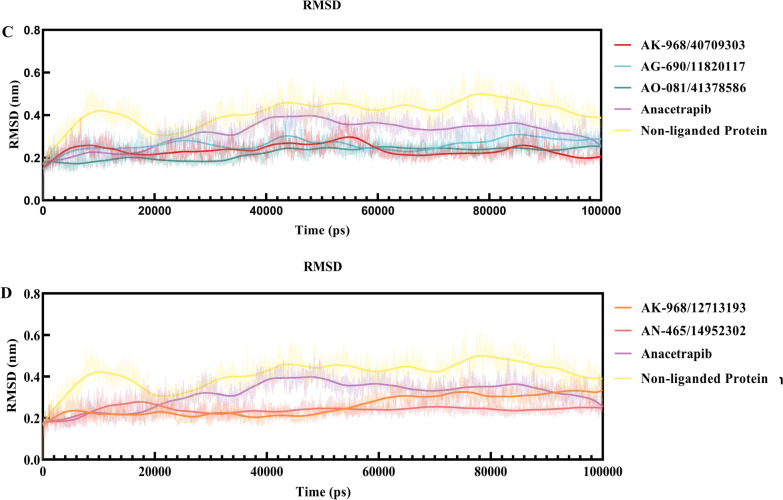
**A** and **B** Root mean squared deviation (RMSD) plots of each ligand, including anacetrapib and five inhibitor molecules, selected for the 100 ns trajectory. **C** and **D** RMSD plots based on Cα atoms of non-ligand protein (PDB ID:2OBD), anacetrapib, and selected inhibitor-bound forms of cholesteryl ester transfer proteins (CETPs) during the duration of 100 ns of molecular dynamics (MD) simulations

MD simulations were performed for the top five compounds identified in the CETP inhibition assay. Trajectory visualization revealed stable complexes between CETP and AK-968/40709303, AG-690/11820117, AO-081/41378586, AK-968/12713193, or AN-465/14952302, using anacetrapib as the reference compound. The ligand-binding pocket of CETP maintained a strong bond with the compounds, indicating no separation of the complex. The RMSD, which measures the average atomic displacement, remained constant throughout the 100 ns trajectory for the protein backbone and ligand, indicating complex stability (Fig. 5). Equilibrium was achieved with RMSD fluctuations of below 2.0 Å for all systems. Based on RMSD, RMSF, radius of gyration (Rg), and molecular mechanics Poisson–Boltzmann surface area (MM-PBSA) calculations, all selected compounds showed the potential to fit within the active site of CETP and form stable bonds throughout the simulation period.

**Fig. 6:**
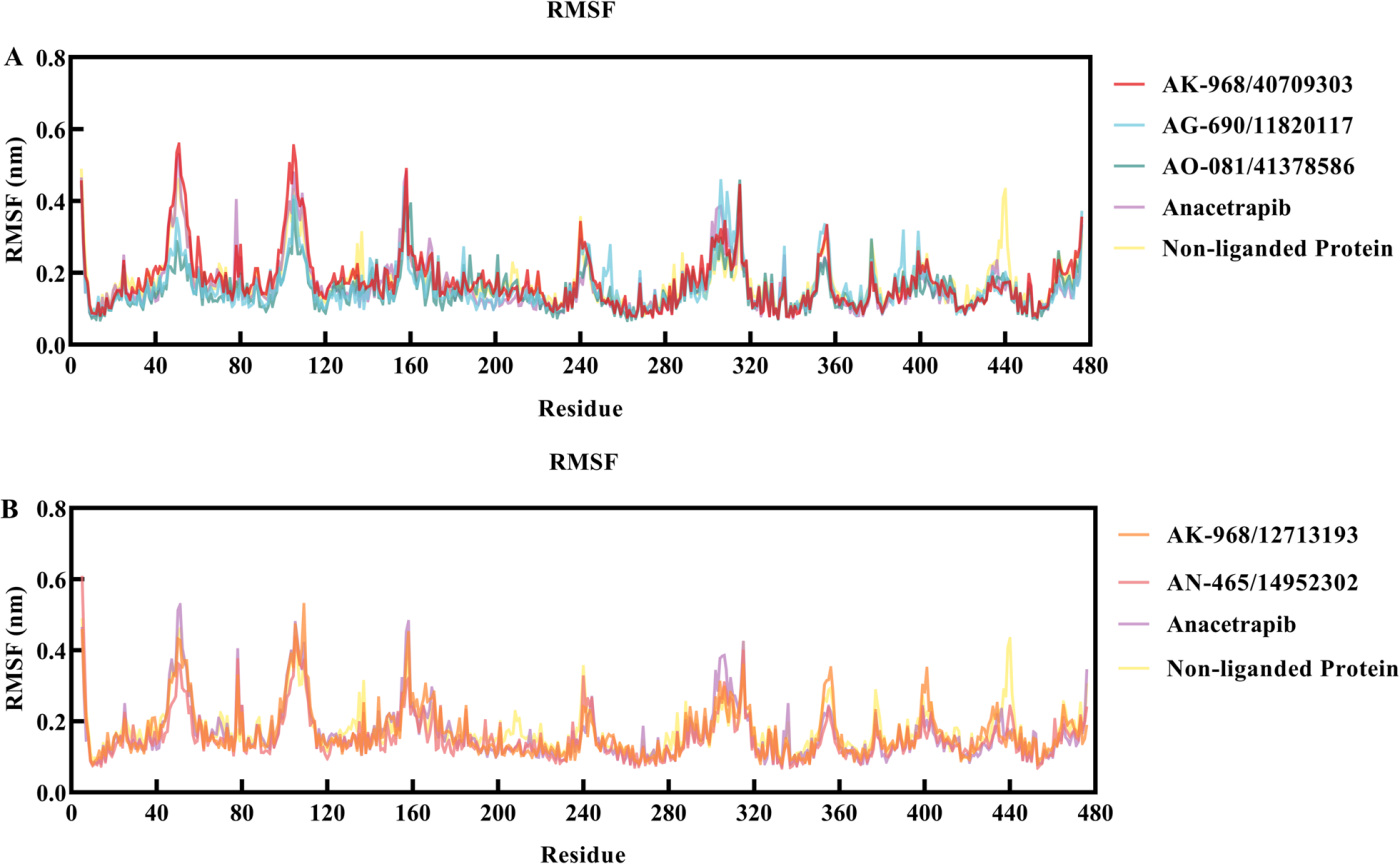
**A** and **B** Per residue, root mean square fluctuation (RMSF) plots of Cα atoms in non-ligand protein (PDB ID:2OBD), anacetrapib, and selected inhibitor-bound forms of cholesteryl ester transfer proteins (CETPs) in 100 ns of molecular dynamics (MD) simulations

The RMSF value served as a metric for assessing the overall flexibility during the MD simulation. Additionally, specific protein residues that interacted with the ligands were evaluated. The RMSF patterns of all the systems exhibited trends comparable to the RMSD fluctuations, with slight fluctuations observed for some residues distant from the active site (Fig. 6). Anacetrapib and the selected inhibitor–CETP complexes showed similar RMS fluctuations. The most fluctuating residues for anacetrapib were Leu440, Ser439, Glu78, Asp240, Phe305, Arg137, Glu157, Gly437, and Arg135, whereas those for the selected inhibitor–CETP complexes were Leu440, Ser439, and Trp106.

**Fig. 7:**
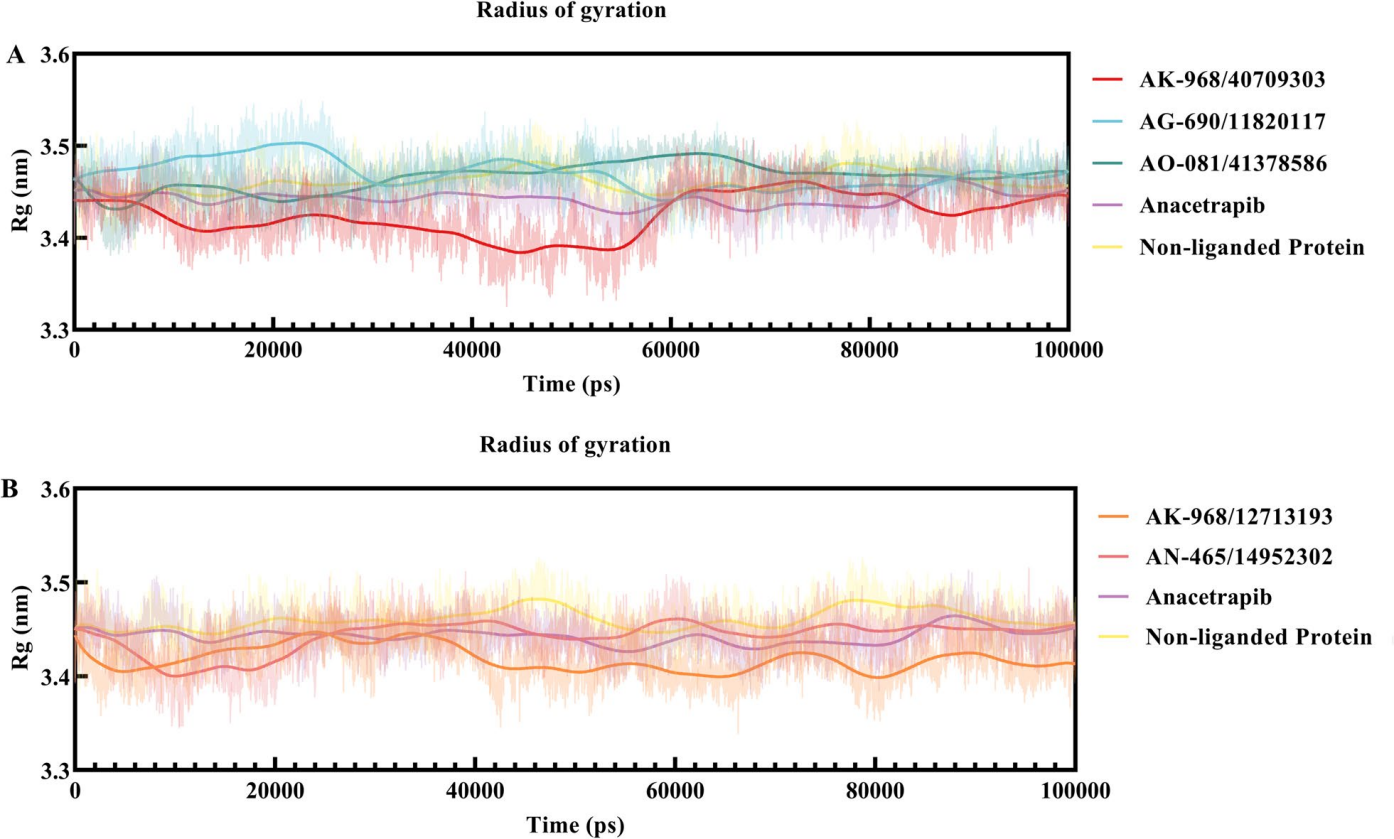
**A** and **B** Radius of gyration (Rg) graph of anacetrapib and selected inhibitor–CETP complexes, along with non-ligand protein (PDB ID:2OBD), during the 100 ns molecular dynamics (MD) simulations

The Rg parameter was calculated to examine the compactness of CETP in the presence of the inhibitors. An inverse relationship existed between stability and Rg or compactness. For example, increased stability correlated with decreased Rg or enhanced compactness, whereas decreased stability was associated with elevated Rg or reduced compactness (Fig. 7). In the unbound state simulation, the CETP protein exhibited no significant unfoldment, as supported by the Rg (unbound) value of 3.46 ± 0.02 nm, which was comparable to that of Rg (co-crystal) (3.45 ± 0.02 nm). For CETP complex with AK-968/40709303, AG-690/11820117, AO-081/41378586, AK-968/12713193, and AN-465/14952302, the Rg values were 3.44 ± 0.02, 3.46 ± 0.02, 3.47 ± 0.01, 3.41 ± 0.02, and 3.45 ± 0.02 nm, respectively (Table 4). The Rg results revealed that all complexes exhibited compactness throughout the simulation, with the hit complexes demonstrating Rg values similar to those of the reference compound.

**Table 4:** Average RMSD, RMSF and Radius of gyration (Rg) of Anacetrapib and selected inhibitors–CETP complexes, along with Non-ligand protein (PDB ID: 2OBD) over 80–100 ns

Compounds	Average RMSD Complexes (nm)	Average RMSD Ligands (nm)	Average RMSF Complexes (nm)	Rg (nm)
AK-968/40709303	0.31 ± 0.03	0.24 ± 0.03	0.18 ± 0.08	3.44 ± 0.02
AG-690/11820117	0.35 ± 0.03	0.25 ± 0.02	0.16 ± 0.07	3.46 ± 0.02
AO-081/41378586	0.31 ± 0.02	0.34 ± 0.02	0.15 ± 0.06	3.47 ± 0.01
AK-968/12713913	0.36 ± 0.03	0.07 ± 0.02	0.17 ± 0.07	3.41 ± 0.02
AN-465/14952302	0.37 ± 0.01	0.26 ± 0.01	0.15 ± 0.06	3.45 ± 0.02
Anacetrapib	0.39 ± 0.04	0.39 ± 0.02	0.16 ± 0.08	3.45 ± 0.02
Non-liganded Protein			0.18 ± 0.07	3.46 ± 0.02

This study calculated the binding free energies of CETP inhibitors to investigate their affinity. The binding energy values were obtained by analyzing the conformations generated through MD simulations at approximately 90–100 ns. The MM-PBSA calculations yielded computed free energies encompassing the electrostatic, van der Waals, nonpolar, and polar solvation energies (Table 5). The MM-PBSA calculations for each system determined the binding strength of each ligand to the active pocket of CETP by analyzing the energy change between the inhibited systems. All five compounds exhibited binding affinities comparable to those of the reference standard.

**Table 5:** Binding free energy calculation of Anacetrapib and selected inhibitor–CETP complexes

Complex	MM-PBSA Calculations (All units kcal/mol) Differences (Complex—Receptor—Ligand)					
	ΔVDWAALS	ΔEPB	ΔENPOLAR	ΔGGAS	ΔGSOLV	ΔTOTAL
AK-968/40709303	− 31.25 ± 2.5	13.38 ± 1.94	0.36 ± 0.15	− 31.25 ± 2.5	13.74 ± 1.85	− 17.51 ± 2.14
AG-690/11820117	− 39.32 ± 3.04	18.62 ± 2.62	0.59 ± 0.23	− 39.32 ± 3.04	19.21 ± 2.54	− 20.1 ± 3.4
AO-081/41378586	− 19.24 ± 3.64	7.75 ± 1.69	1.44 ± 0.29	− 19.24 ± 3.64	9.19 ± 1.5	− 10.06 ± 3.08
AK-968/12713193	− 30.02 ± 2.16	15.74 ± 2.34	− 0.19 ± 0.08	− 30.02 ± 2.16	15.55 ± 2.32	− 14.47 ± 2.5
AN-465/14952302	− 42.77 ± 2.13	18.64 ± 1.34	0.2 ± 0.15	− 42.77 ± 2.13	18.84 ± 1.4	− 23.94 ± 2.81
Anacetrapib	− 36.35 ± 2.34	17.46 ± 1.54	0.91 ± 0.14	− 36.35 ± 2.34	18.37 ± 1.53	− 17.98 ± 2.51

ΔVDWAALS: van der Waals free energy; ΔEPB: polar component of solvation-free energy; ΔENPOLAR: the non-polar component ofthe solvation energy; ΔGGAS: the gas-phase molecular mechanics free energy; ΔGSOLV: the solvation free energy; ΔTOTAL: total binding free energy

**Fig. 8:**
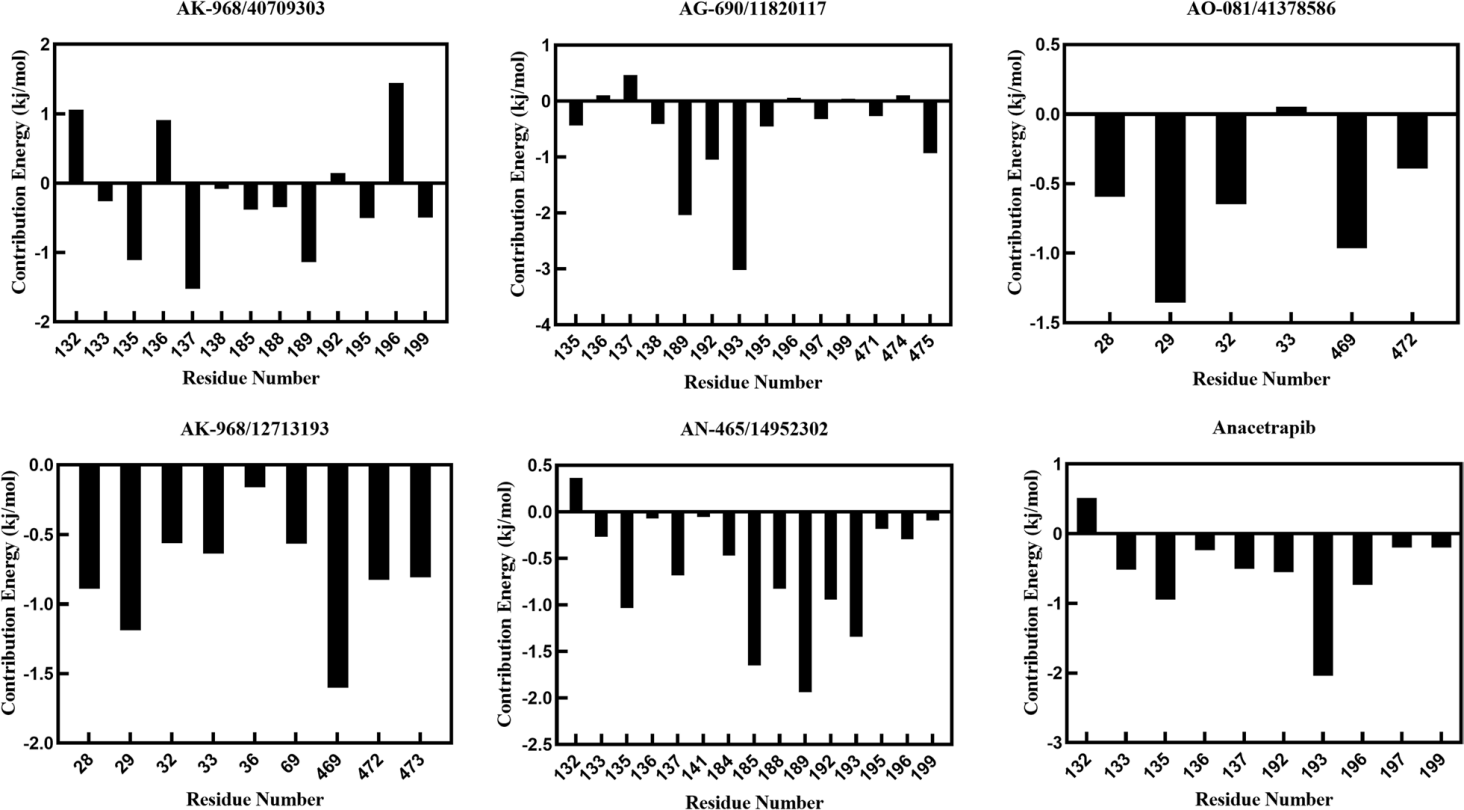
Per-residue binding free energy decomposition of protein − ligand complexes

The binding free energy was subsequently decomposed, and the individual residue-binding free energy was calculated to elucidate the influence of each residue on the overall binding energy. Residues with interaction energies below –1 kcal/mol were considered "hot residues" crucial for binding. For CETP-anacetrapib, only Ile 193 exhibited an interaction energy with the ligand below the threshold. In CETP-AK-968/40709303, three residues, Arg135, Arg137, and Val189, contributed significantly to ligand binding. For CETP-AG-690/11820117, three residues (Val189, Asn192, and Ile193) contributed to the higher energy values. In CETP-AO-081/41378586, only Lys29 exhibited notably higher energy values. Two residues (Lys29 and Val469) are considered vital for CETP-AK-968/12713193. Additionally, four residues (Arg135, Lys185, Val189, and Ile193) displayed the highest energy values (Fig. 8) for CETP-AN-465/14952302. Notably, Lys29, Arg135, Val189, and Ile193 residues interacted with two or more inhibitors. This implies that these four residues may serve crucial stabilizing roles in the pocket architecture and favorably orient the ligands, thereby contributing to the inhibitory activity. A meaningful finding is the importance of these positions, identified through a comparative analysis of engagement patterns across the chemical series. Targeted mutagenesis of the implicated residues in future studies could help validate their proposed functions in maintaining complex stability. Overall, the computational investigation enhances the structural understanding of CETP inhibition and may guide the continued optimization of selective pharmacological agents.

To the best of our knowledge, the CETP inhibition activities of the five compounds identified in this study have not been previously reported. While recent studies [33–35] have utilized molecular docking to investigate binding patterns and affinities toward CETP, and pharmacophore mapping has been employed to study CETP inhibitors [35], none have utilized MD simulations to evaluate screened inhibitors. Our study introduces a novel approach by combining 3D-QSAR pharmacophore modeling, molecular docking, and MD simulations to screen CETP inhibitors in the Specs database.

In terms of clinical relevance, the identification of these potential CETP inhibitors holds promise for the development of therapies targeting atherosclerotic diseases. Given the critical role of CETP in lipid metabolism and its association with cardiovascular disorders, compounds exhibiting CETP inhibition activity could potentially mitigate atherosclerosis progression. However, it's essential to acknowledge that further experimental validation and clinical investigation are imperative to determine the therapeutic efficacy and safety profile of these compounds in clinical settings. Our study serves as a foundational step towards translating computational findings into clinically relevant interventions for cardiovascular diseases.

## Conclusions

This study employed 3D-QSAR pharmacophore modeling and AutoDock Vina molecular docking to screen 26 CETP inhibitors. Subsequent 100 ns MD simulations on AK-968/40709303, AG-690/11820117, AO-081/41378586, AK-968/12713193, and AN-465/14952302 revealed the stability of CETP complexes and efficacy of the identified compounds. Inhibition assay results further validated their effectiveness. Investigation into the mode of action under simulated physiological conditions provided insight into their potential mechanisms. However, these findings necessitate further experimental validation and clinical investigation for their potential in treating atherosclerotic diseases. Our study contributes to the field by employing a comprehensive computational approach to identify potential CETP inhibitors. Future research should focus on validating these compounds experimentally and exploring their therapeutic potential through clinical trials. This work paves the way for the development of novel treatments for cardiovascular disorders.

### Supplementary Information

**Additional file 1:** **Fig S1.** Protein-ligand interactions of binding mode between CETP and hits. **Fig S2.** Screening assay of identified compounds as novel CETP inhibitors in vitro. Inhibitory activity of the 21 inhibitor molecules against CETP. **Table S1.** Physiochemical properties of 26 compounds calculated by SwissADME and ADMETlab 2.0 . **Table S2.** Docking results of CETP and the selected hits from the docking-based virtual screening stating the hydrogen bonds and hydrophobic interactions. **Table S3.** Screening assay of identified compounds as novel CETP inhibitors in vitro.

## Data Availability

All data generated or analysed during this study are included in the manuscript.
