# Correction: Discovery of novel cholesteryl ester transfer protein (CETP) inhibitors by a multi‑stage virtual screening

**DOI:** 10.1186/s13065-024-01222-2

**Published:** 2024-07-05

**Authors:** Yanfeng Liu, Liangying Deng, Feng Ding, Qiang Wang, Shuran Zhang, Nana Mi, Wenhui Zhang, Bailin Zeng, Huangjin Tong, Lixing Wu

**Affiliations:** 1 Nanjing Lishui District Hospital of Traditional Chinese Medicine, Nanjing, China; 2 Affiliated Hospital of Integrated Traditional Chinese and Western Medicine, Nanjing University of Chinese Medicine, Nanjing, China; 3 School of Basic Medicine and Clinical Pharmacy, China Pharmaceutical University, Nanjing, China

**Correction to: BMC Chemistry (2024) 18:95** 10.1186/s13065-024-01192-5

Following publication of the original article [1], the authors reported two errors. In graphical abstract, AK-968/12713193 was incorrectly written as AQ-432/43400141. The corrected version of the graphical abstract has now been shown below.

Graphical abstract
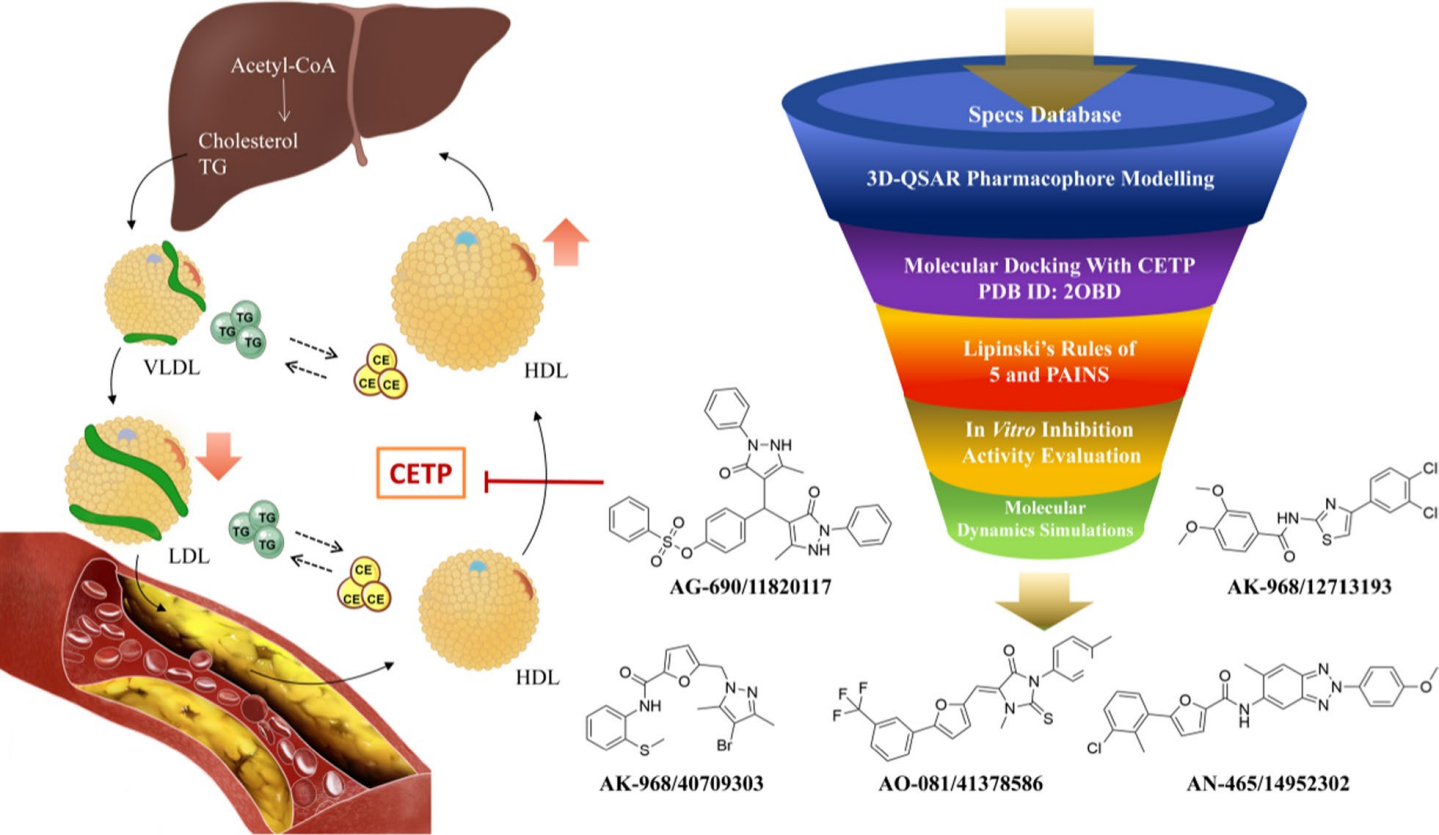


In Results and Discussion, the unit “− 1 kcal/mol” was incorrectly written as “1 kcal/mol” in the second sentence of the fifth paragraph under MD simulations section.

The original article has been corrected.
