# Supplementary material for: Discovery of novel cholesteryl ester transfer protein (CETP) inhibitors by a multi-stage virtual screening
Source: BMC Chem. 2024 May 3;18(1):95. doi: 10.1186/s13065-024-01192-5 (PMC11069292; doi:10.1186/s13065-024-01192-5)

**Additional file 1**

**Fig S1** Protein-ligand interactions of binding mode between CETP and hits

**Fig S2** Screening assay of identified compounds as novel CETP inhibitors in vitro. Inhibitory activity of the 21 inhibitor molecules against CETP

**Table S1** Physiochemical properties of 26 compounds calculated by SwissADME and ADMETlab 2.0

**Table S2** Docking results of CETP and the selected hits from the docking-based virtual screening stating the hydrogen bonds and hydrophobic interactions

**Table S3** Screening assay of identified compounds as novel CETP inhibitors in vitro

**Table S1** physiochemical properties of 26 compounds calculated by SwissADME and ADMETlab 2.0

| Compounds Number | Mol weight | LogP | H-bond acceptors | H-bond donors | PAINS |
| --- | --- | --- | --- | --- | --- |
| AK-968/40709303 | 420.33 | 4.098 | 3 | 1 | 0 |
| AG-690/11820117 | 592.67 | 4.466 | 5 | 2 | 0 |
| AG-690/13702182 | 497.42 | 6.216 | 4 | 1 | 0 |
| AK-968/41024609 | 498.81 | 5.533 | 4 | 2 | 0 |
| AN-153/14955286 | 450.88 | 5.399 | 5 | 2 | 0 |
| AP-970/43483336 | 479.95 | 5.34 | 6 | 1 | 0 |
| AP-970/43337008 | 481.93 | 4.441 | 5 | 1 | 0 |
| AN-023/13853317 | 326.78 | 3.959 | 3 | 1 | 0 |
| AN-988/41010304 | 439.47 | 4.98 | 4 | 1 | 0 |
| AG-670/12353018 | 476.31 | 6.335 | 4 | 1 | 0 |
| AO-081/41378586 | 456.49 | 5.722 | 5 | 0 | 0 |
| AN-329/14660014 | 357.38 | 1.923 | 4 | 3 | 0 |
| AT-057/43313916 | 464.52 | 2.571 | 6 | 0 | 0 |
| AQ-390/42134831 | 466.6 | 5.58 | 4 | 2 | 0 |
| AK-968/15607179 | 438.71 | 4.266 | 4 | 1 | 0 |
| AK-968/40708741 | 443.67 | 3.983 | 5 | 1 | 0 |
| AK-968/41922861 | 476.51 | 3.214 | 6 | 1 | 0 |
| AQ-432/43400115 | 498.34 | 3.533 | 6 | 2 | 0 |
| AK-968/40709316 | 481.98 | 4.87 | 5 | 2 | 0 |
| AK-968/41022657 | 343.82 | 2.177 | 3 | 1 | 0 |
| AH-487/15582195 | 523.65 | 4.284 | 5 | 0 | 0 |
| AO-022/43455311 | 470.88 | 3.396 | 6 | 2 | 0 |
| AQ-432/43400141 | 406.9 | 2.955 | 6 | 2 | 0 |
| AK-968/12713193 | 409.29 | 4.371 | 4 | 1 | 0 |
| AO-022/43514021 | 414.46 | 1.925 | 5 | 3 | 0 |
| AN-465/14952302 | 472.93 | 6.417 | 5 | 1 | 0 |

Note: PAINS: Pan Assay interference compounds, If a molecule is labeled 0, no alarm is displayed in PAINS

**Table S2** Docking results of CETP and the selected hits from the docking-based virtual screening stating the hydrogen bonds and hydrophobic interactions

| Compounds Number | Binding affinities (Kcal/mol) | Residues Interactions of hydrogen bonds | Residues of hydrophobic interactions |
| --- | --- | --- | --- |
| AK-968/40709303 | -6.3 | R137, N192, V189 | N188, N192, D196 |
| AG-690/11820117 | -7.8 | R137, N192 | N188, V189, N192, D196 |
| AG-690/13702182 | -7.3 | Not Applicable | L475, F471, V189, I193, D196, R137, R135 |
| AK-968/41024609 | -6.8 | T450, R451, Q372 | Q372, E447 |
| AN-153/14955286 | -7.3 | V189, N188 | R135, D196, V189/N188, K185 |
| AP-970/43483336 | -8.2 | K185, N188, R137, N192 | D196, N192, N188 |
| AP-970/43337008 | -7.3 | R135, S216, D214, V213, D240, S207 | Not Applicable |
| AN-023/13853317 | -7.4 | D470, S474 | I193, F197, F471, L467, D470 |
| AN-988/41010304 | -8.5 | S72, Q32 | A28, V469 |
| AG-670/12353018 | -8.4 | K29 | V469 |
| AO-081/41378586 | -7.5 | I69 | N24, A28, K29, V469 |
| AN-329/14660014 | -7.2 | Q23, A28, K29 | K29 |
| AT-057/43313916 | -6.4 | S242 | I237 |
| AQ-390/42134831 | -7.8 | A140, N188, N188, V189, R137 | A140, P141, N188, V189, N192 |
| AK-968/15607179 | -6.5 | K185, N188, N192, R137 | N192 |
| AK-968/40708741 | -7.1 | D196, N192, R137, N188, V189 | N188, N192 |
| AK-968/41922861 | -7.6 | D214, I215, S216, R135, D196, N192 | N192, I193 |
| AQ-432/43400115 | -5.6 | V213, S207, G212 | D214, V213, G212 |
| AK-968/40709316 | -7.5 | N192, N188, K185 | V189, N188, K185 |
| AK-968/41022657 | -6.2 | D196, R137, N192, | V189, V189, N188, R135 |
| AH-487/15582195 | -6.1 | R135 | A203, S207, |
| AO-022/43455311 | -6.6 | R135, S207, S216, I215, I215, D214, D214, V213, V213, G212, G212 | I237 |
| AQ-432/43400141 | -7 | V136, R137, N192, D196, I193 | Not Applicable |
| AK-968/12713193 | -6.6 | I69, Q32 | A28, K29, L472 |
| AO-022/43514021 | -7.9 | V189, R137, D196 | R135, D196, R137, N192, N188 |
| AN-465/14952302 | -7.7 | R137, N188 | D196, N192, V189, N188 |

**Table S3** Screening assay of identified compounds as novel CETP inhibitors in *vitro*

| Compounds Number | Inhibition at 10㎛(%) |
| --- | --- |
| AK-968/40709303 | 37.94±6.99 |
| AG-690/11820117 | 28.68±3.05 |
| AG-690/13702182 | 20.50±0.82 |
| AK-968/41024609 | 5.23±3.49 |
| AN-153/14955286 | 23.15±4.29 |
| AP-970/43483336 | 13.74±1.89 |
| AP-970/43337008 | 22.29±4.35 |
| AN-023/13853317 | 23.25±7.49 |
| AN-988/41010304 | 16.38±7.91 |
| AG-670/12353018 | 21.76±0.58 |
| AO-081/41378586 | 25.05±10.34 |
| AN-329/14660014 | 3.64±2.63 |
| AT-057/43313916 | 11.28±27.72 |
| AQ-390/42134831 | 6.87±4.40 |
| AK-968/15607179 | 0.62±6.70 |
| AK-968/40708741 | 3.48±9.32 |
| AK-968/41922861 | -17.26±30.46 |
| AQ-432/43400115 | -6.83±5.97 |
| AK-968/40709316 | -29.21±20.74 |
| AK-968/41022657 | 21.25±12.92 |
| AH-487/15582195 | 22.57±4.76 |
| AO-022/43455311 | 24.99±4.36 |
| AQ-432/43400141 | 1.07±36.45 |
| AK-968/12713193 | 36.88±0.94 |
| AO-022/43514021 | 22.41±4.73 |
| AN-465/14952302 | 33.35±4.40 |


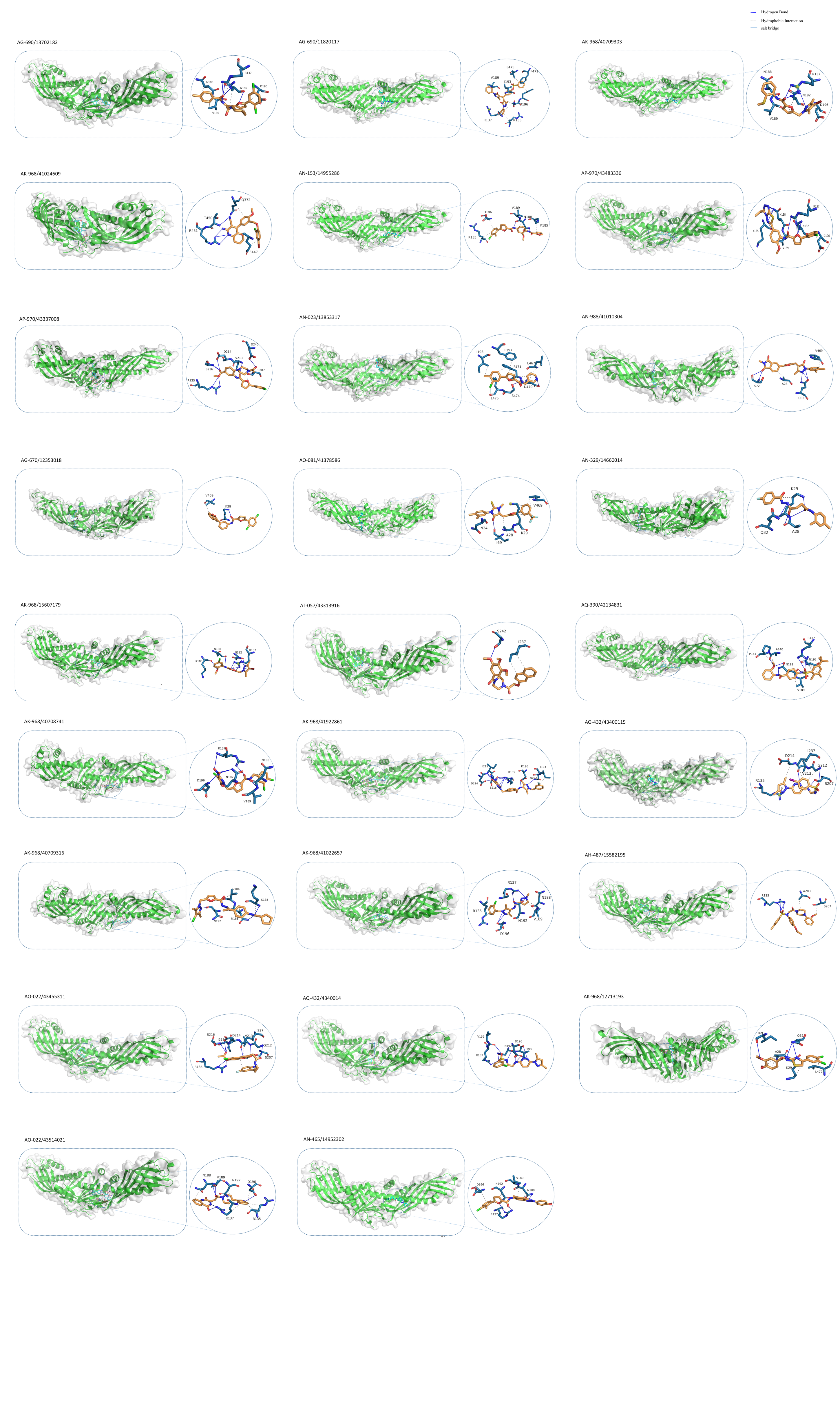


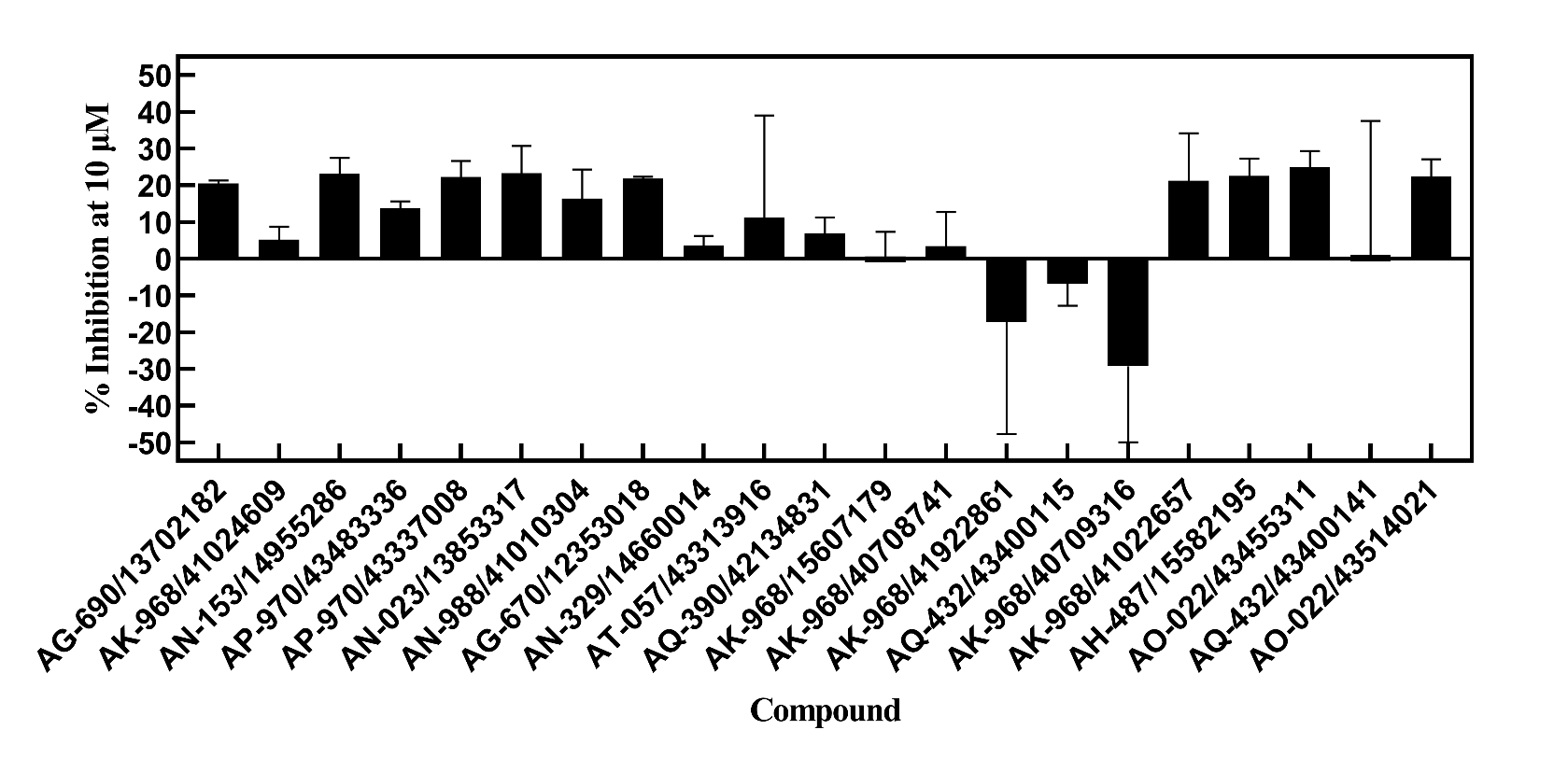

Supplement: Supplementary file 1 — Additional file 1: Fig S1. Protein-ligand interactions of binding mode between CETP and hits. Fig S2. Screening assay of identified compounds as novel CETP inhibitors in vitro. Inhibitory activity of the 21 inhibitor molecules against CETP. Table S1. Physiochemical properties of 26 compounds calculated by SwissADME and ADMETlab 2.0 . Table S2. Docking results of CETP and the selected hits from the docking-based virtual screening stating the hydrogen bonds and hydrophobic interactions. Table S3. Screening assay of identified compounds as novel CETP inhibitors in vitro. [file 13065_2024_1192_MOESM1_ESM.docx]
